# Effect of liposomal formulation of ascorbic acid on corneal permeability

**DOI:** 10.1038/s41598-023-29290-9

**Published:** 2023-03-01

**Authors:** Anita Csorba, Gábor Katona, Mária Budai-Szűcs, Diána Balogh-Weiser, Anna Maria Fadda, Carla Caddeo, Ágnes Ildikó Takács, Péter Mátyus, György T. Balogh, Zoltán Zsolt Nagy

**Affiliations:** 1 Department of Ophthalmology, Semmelweis University, Mária Street 39., 1085 Budapest, Hungary; 2 Institute of Pharmaceutical Technology and Regulatory Affairs, Faculty of Pharmacy, University of Szeged, Eötvös Street 6., 6720 Szeged, Hungary; 3 Department of Physical Chemistry and Materials Science, Faculty of Chemical Technology and Biotechnology, Budapest University of Technology and Economics, Műegyetem Rkp. 3., 1111 Budapest, Hungary; 4 Department of Life and Environmental Sciences, University of Cagliari, Via Ospedale 72, 09124 Cagliari, Italy; 5 E-Group ICT SOFTWARE, Kacsa Street 11, 1027 Budapest, Hungary; 6 Department of Chemical and Process Engineering, Faculty of Chemical Technology and Biotechnology, Budapest University of Technology and Economics, Műegyetem Rkp. 3., 1111 Budapest, Hungary; 7 Institute of Pharmacodynamics and Biopharmacy, Faculty of Pharmacy, University of Szeged, Eötvös Street 6., 6720 Szeged, Hungary

**Keywords:** Biochemistry, Drug delivery

## Abstract

Ascorbic acid (AA) has a pivotal role in corneal wound healing via stimulating the biosynthesis of highly organized extracellular matrix components, but its rapid degradation and low corneal permeability limits its therapeutic effects. In this paper, we present the pharmacokinetic properties of a liposomal-based formulation of AA in terms of corneal permeation. Chemical stability, shelf-life, and drug release rate of lyophilized liposome (AA-LLipo) formulation was determined in comparison to free-form of AA solution using high-performance liquid chromatography (HPLC) and rapid equilibrium dialysis. In vitro transcorneal permeability was studied using a parallel artificial membrane permeability assay (PAMPA). Ex vivo permeation was examined on AA-LLipo-treated porcine cornea by determining the AA content on the ocular surface, in the cornea as well as in the aqueous humor using HPLC, and by Raman-mapping visualizing the AA-distribution. Our results showed that the liposomal formulation improved the chemical stability of AA, while drug release was observed with the same kinetic efficiency as from the free-form of AA solution. Both corneal-PAMPA and porcine corneal permeability studies showed that AA-LLipo markedly improved the corneal absorption kinetics of AA, thus, increasing the AA content in the cornea and aqueous humor. AA-LLipo formulation could potentially increase the bioavailability of AA in corneal tissues.

## Introduction

Ascorbic acid (AA) is a natural, water-soluble vitamin, which plays an essential role in several physiologic and metabolic functions in the human body. Due to the lack of the enzyme—ʟ-gulonolactone oxidase—which is responsible for the last step of AA synthesis, it cannot be endogenously synthesized or stored in humans, thus, dietary intake is essential^1^. Since AA is a major antioxidant molecule, it protects tissues against free radicals, which are formed during metabolism and associated with diseases and injuries^2^.

Acting as a co-factor in hydroxylation, AA plays an important role in collagen biosynthesis^3^. Several studies have shown that the presence of AA can stimulate the biosynthesis of extracellular matrix (ECM) components and fibroblast proliferation^4–6^. This process has also been demonstrated in human fibroblasts^7^. Moreover, Guo et al. showed that AA-stimulated human corneal fibroblasts tend to produce highly regular ECM fibrils, which indicates that AA has a crucial role in wound healing of corneal tissues and in mitigation of scar formation^8^. In an in vitro study, Chen et al. found that AA promotes the activation of corneal epithelial stem cells and progenitor cells as well^9^. It has been documented that ocular tissues contain far more AA than other tissues in diurnal species^10^. Brubaker et al. reported that the corneal epithelium has the highest AA contents among all reported tissues, which indicates its protective role against photooxidative damage in order to defend deeper ocular tissues from UV-radiation^10^. Interestingly, it has been shown that the concentration of AA in the corneal epithelium was the highest in the central, pupillary region, supporting that it has an important role in UV-filter function^11^. There is evidence that suggests that AA can promote epithelial healing also in vivo^9^. In clinical practice, the beneficial effects of topical AA therapy have been shown in the treatment of alkali burns^12^. Both oral and topical AA-supplementation could reduce postoperative stromal opacification after excimer laser photoablation^3, 13^. Therapeutic benefits of AA have been shown both in corneal neovascularization and in infectious keratitis^14, 15^.

Thus, according to the recently available literature, increased concentrations of AA might have beneficial effects on the wound healing process of the cornea. Corneal absorption from the ocular surface represents the main mechanism of absorption of topically inserted drugs. However, lipophilic properties of the corneal epithelial membrane serve as a diffusion barrier against hydrophilic substances, thus, permeability of the polar AA through the predominantly lipophilic corneal epithelium might be insufficient. It has been reported that increasing the free AA concentration at the precorneal area results in an increased permeated AA through the cornea, although after further increase, permeation may reach a plateau^16^. Moreover, aqueous solution of free-form AA is unstable at room temperature and to light exposure^17^. AA is degradable in aqueous environment, which can be promoted at high pH, in presence of metal ions and oxygen. The main strategies to overcome this degradation process can be the selection of more stable AA derivatives; to control or eliminate the presence of oxygen in the container; reduction of water content (application of anhydrous/nonaqueous formulations); to use low pH formulations, antioxidants, anti-chelating agents^18–20^ or to apply micro-, or nanoencapsulated drug delivery systems^20, 21^. Nano-, and microparticulate drug delivery systems can be used to increase both the stability and permeability of AA, and thus the bioavailability of AA. Among others, chitosan based^22^ or mesoporous nanoparticles^23^, solid lipid nanoparticles^24^, microemulsions and micelles^25^ can be found in the literature as carriers of AA applied via different administration routes.

Recently, liposomal encapsulation of AA has been used through oral administration, since it can promote the protection and activity of encapsulated compounds^26^. However, liposomal formulation of AA in ophthalmic usage has not been studied yet. This research aimed to evaluate the stability and corneal permeation ability of liposomal AA formulation using in vitro corneal parallel artificial membrane permeability assay (PAMPA) and ex vivo porcine cornea.

## Materials

Soy phosphatidylcholine (> 90%; P90G) was purchased from Lipoid GmbH (Ludwigshafen, Germany). Ascorbic acid (AA), disodium EDTA, and sodium sulfite (Na_2_SO_3_) were purchased from Galeno (Carmignano, Prato, Italy). Phosphate buffer (PBS, pH 7.4) was purchased from Sigma-Aldrich/Merck (Milan, Italy). Hydrocortisone, buspirone, potassium dihydrogen phosphate (K_2_HPO_4_) and L-α-phosphatidylcholine (PC) were purchased from Sigma Aldrich Co. Ltd. (Budapest, Hungary). The analytical grade solvents acetonitrile, hexane, dodecane and chloroform were purchased from Merck KGaA (Darmstadt, Germany).

### Methods

This study was carried out with the approval of the Semmelweis University Regional and Institutional Committee of Sciences and Research Ethics (SE RKEB: 51/2021). All experiments were performed in accordance with relevant guidelines and regulations.

### Preparation and characterization of lyophilized liposomes

Liposomes containing ascorbic acid (AA-Lipo) were made from soy phosphatidylcholine (60 mg/mL), ascorbic acid (50 mg/mL), disodium EDTA (0.025% w/v), and sodium sulfite (0.1% w/v). The components were weighed in a vial, 2 mL of PBS was added, and the dispersion was sonicated (30 cycles, 5 s on and 2 s off; 13 microns of probe amplitude) with a Soniprep 150 plus ultrasonic disintegrator (MSE Crowley, London, UK). The samples were prepared and kept in the dark during the experimental time.

The mean diameter, polydispersity index (P.I., a measure of the width of size distribution), and zeta potential of the AA-Lipo samples were determined by dynamic and electrophoretic light scattering using a Zetasizer nano-ZS (Malvern Panalytical, Worcestershire, UK). Samples (*n* = 300) were diluted with PBS (1:100) and analyzed at 25 °C. The liposome dispersions were filtered (0.20 μm) and the mean diameter, P.I., and zeta potential were measured again.

The sterile filtered AA-Lipo samples were frozen at − 80 °C and freeze-dried for 24 h at − 90 °C in an FDU-8606 freeze-drier (Operon, Gimpo, Korea). The lyophilized liposome (AA-LLipo) samples were re-hydrated with sterile water (2 mL) and the mean diameter, P.I., and zeta potential were analyzed.

The rheological behavior of AA-LLipo liposomal formulation and standard AA (API) solution was investigated by the determination of dynamic viscosity using a Physica MCR 301 rheometer (Anton Paar, Graz, Austria). A probe with a cone-plate geometry (with a diameter of 25 mm and cone angle of 1°) was used for the measurements. The measuring chamber of the instrument was thermostated at 35.0 °C according to the temperature of corneal surface. For each sample the applied interval of shear rate was 1–1000 s^−1^. Six parallel measurements were performed, data is presented as mean ± SD.

### Chemical stability and shelf-life determination

For chemical stability investigation, solutions with 140 mM target concentration of standard AA (API) and AA-LLipo were prepared in pH 7.4 PBS. The solutions were incubated at 35 °C on an orbital shaker (Heidolph Titramax 1000, Heidolph Instruments, Schwabach, Germany) at 350 rpm for 4 h. Samples were withdrawn at 0, 10, 20, 30, 60, 120, 180 and 240 min and AA concentrations were determined by high-performance liquid chromatography with diode-array detection (HPLC–DAD). Five parallel measurements were performed, data is presented as mean ± SD. Shelf-life was determined based on the 10% decrease in AA content in comparison to target concentration.

### Rapid equilibrium dialysis (RED)

The RED Device (Thermo Scientific™, Waltham, Massachusetts, USA) was used for the determination of time-dependent release profiles of AA from AA-LLipo formulation in comparison to AA (API) solution. AA-LLipo formulation was re-dispersed in 2 mL PBS (pH 7.4) and homogenized by using an Eppendorf MixMate vortex mixer for 30 s and by an ultrasonic bath (Bandelin Sonorex Digiplus) for 10 min. The RED Device inserts (8 K MWCO) were fitted into reusable Teflon base plate, then 100 µL of samples were placed into the donor chambers. Thereafter, 300 µL of PBS (pH 7.4) was added to the acceptor chambers, the unit was covered with a sealing tape and incubated at 35 °C on an orbital shaker at 350 rpm for 4 h. Samples were withdrawn at 5, 15, 30, 60, 120 and 240 min from the acceptor chambers and AA concentrations were determined by HPLC–DAD. Five parallel measurements were performed, data is presented as mean ± SD.

### High performance liquid chromatography (HPLC)

The determination of AA concentration was performed with HPLC using an Agilent 1260 (Agilent Technologies, Santa Clara, USA). As stationary phase a Kinetex® EVO C18 column (5 µm, 150 mm × 4.6 mm (Phenomenex, Torrance, CA, USA) was applied. Isocratic elution with purified water adjusted to pH 3.0 with phosphoric acid and acetonitrile 80:20 (v/v) was applied for 3 min at a flow rate of 1.0 mL/min at 30 °C. 10 µL of the samples were injected to determine the AA concentration. The chromatograms were detected at 254 nm using UV–VIS diode array detector. Data was evaluated using ChemStation B.04.03. Software (Agilent Technologies, Santa Clara, USA). The retention time of AA was at 1.56 min. A five-point calibration curve was generated using a linear regression analysis of the peak areas were plotted against known concentrations of AA in the range of 1.5–1500 µg/mL. Each time point per formulation was measured in triplicate. The linear regression of the calibration line was 0.9998, the limit of detection (LOD) and quantification (LOQ) was 1.5 ppm and 5 ppm, respectively.

### In vitro corneal permeability measurements

Parallel artificial membrane permeability assay (PAMPA) was used to determine the transcorneal permeability of AA from the reference solution and the re-dispersed AA-LLipo formulations. The filter donor plate (Multiscreen™-IP, MAIPN4510, pore size 0.45 µm; Millipore, Merck Ltd., Budapest, Hungary) was coated with 5 µL of different concentration of L-α-phosphatidylcholine (2.2, 8.9, 17.8 and 26.7 mg/mL) dissolved in a solvent mixture (70% (v/v) hexane, 25% (v/v) dodecane, 5% (v/v) chloroform. The Acceptor Plate (MSSACCEPTOR; Millipore, Merck Ltd., Budapest, Hungary) was filled with 300 μL of a pH 7.4 PBS. 150–150 μL of the formulation and the reference solutions (100 µM hydrocortisone, 100 µM buspirone and 100 µM AA) were transferred on the membrane of the donor plate. Then, the latter was covered with a plate lid in order to decrease the possible evaporation of the solvent. This sandwich system was incubated at 35 °C for 4 h. The concentration of AA permeated in the acceptor plate was determined by HPLC. The effective permeability and membrane retention of AA was calculated using the following equation ^27^:

1 $${P}_{e}= \frac{-2.303}{A \cdot (t-{\tau }_{ss})} \cdot \left(\frac{1}{1+{r}_{v}}\right) \cdot \mathrm{lg}\left[-{r}_{v}+\left(\frac{1+{r}_{v}}{1-MR}\right) \cdot \frac{{c}_{D}\left(t\right)}{{c}_{D}\left(0\right)}\right]$$

where *P*_*e*_ is the effective permeability coefficient (cm/s), *A* is the filter area (0.3 cm^2^), *t* is the incubation time (s), *τ*_*ss*_ is the time to reach steady-state (s), *r*_*v*_ is the volume ratio of aqueous compartments (*V*_*D*_*/V*_*A*_), *V*_*D*_ and *V*_*A*_ are the volumes in the donor (0.15 cm^3^) and acceptor phase (0.3 cm^3^), *c*_*D*_*(t)* is the concentration of the compound in the donor phase at time point *t* (mol/cm^3^), *c*_*D*_*(0)* is the concentration of the AA in the donor phase at time point zero (mol/cm^3^) and MR is the membrane retention factor, defined as follows ^16^:

2 $$MR=1-\frac{{c}_{D}\left(t\right)}{{c}_{D}\left(0\right)}-\frac{{V}_{A}{c}_{A}(t)}{{V}_{D}{c}_{D}(0)}$$

where *c*_*A*_*(t)* is the concentration of AA in the acceptor phase at time point *t* (mol/cm^3^). Six parallel measurements were performed, data is presented as mean ± SD.

### Ex vivo permeation study on porcine cornea

The ex vivo penetration of AA was examined as follows: a porcine eye was treated with the formulations (i.e., AA aqueous solution or re-dispersed AA-LLipo). Then, the residual AA concentration on the corneal surface, the adsorbed AA concentration in the cornea, and penetrated amount of AA into the aqueous humor was measured with HPLC, and the distribution of the AA was visualized by Raman mapping.

Freshly donated porcine eyes obtained from a local slaughterhouse was placed on a sterile cotton wool bed moistened with physiological saline solution and kept in refrigerator box during the transportation. The porcine eye was placed into a Teflon cell, where the cornea was uncovered and surrounded by a Teflon ring to prevent the flow out of eye drops. The cornea was instilled with 1000 μL of physiological saline solution and 50 μL of liquid formulation was added to the covering saline solution, which can mimic physiological dilution caused by tear fluid. The eye was incubated at 35 °C. After 15, 30 and 60 min, individual treatment residual solution from the topical surface and the aqueous humor was aspirated through corneal paracentesis 2–5 h postmortem. The cornea was excised and AA was extracted with 2 mL methanol:water 50:50 (v/v) using an orbital shaker (PSU-10i Orbital Shaker, Grant Instruments Ltd, Cambs, England) for 60 min, at 450 rpm. The corneal retention (MR) of AA, the apparent permeability (P_app_C) of AA into the cornea and the apparent permeability (P_*app*_Aq) of AA into the aqueous humor was calculated by using Eqs. (3), (4) and (5), respectively:

3 $$MR=1-\frac{{c}_{CS}\left(t\right)}{{c}_{CS}\left(0\right)}-\frac{{V}_{AC}{c}_{C}(t)}{{V}_{CS}{c}_{CS}(0)}$$

4 $${P}_{app}C(cm/s)=\frac{\Delta {\left[C\right]}_{Aq}\times {V}_{AC}}{A\times {\left[C\right]}_{CS}\times \Delta t}$$

5 $${P}_{app}Aq(cm/s)=\frac{\Delta {\left[C\right]}_{C}\times {V}_{AC}}{A\times {\left[C\right]}_{CS}\times \Delta t}$$

where *c*_*CS*_*(t)* is the concentration of AA on the corneal surface at time point *t* (mol/cm^3^), *c*_*CS*_*(0)* is the concentration of AA on the corneal surface at time point zero (mol/cm^3^), *c*_*C*_*(t)* is the concentration of AA in the cornea at time point *t* (mol/cm^3^), *V*_*AC*_ and *V*_*CS*_ are the volumes in the anterior chamber (0.25 cm^3^) and on the corneal surface (1.05 cm^3^).

P_*app*_C was calculated from the concentration difference of AA in the aqueous humor (*Δ[C]Aq*) after treatment, initial concentration of the compound on the corneal surface at time point zero (*[C]CS*), the volume of anterior chamber *V*_*AC*_ (250 µL), *A* is the surface area available for permeability (1.77 cm^2^) and *t* is the incubation time (s). P_*app*_Aq was calculated from the concentration difference of AA in the cornea (*Δ[C]C*) after treatment, initial concentration of the compound on the corneal surface at time point zero (*[C]CS*), the volume of anterior chamber *V*_*AC*_ (250 µL), *A* is the surface area available for permeability (1.77 cm^2^) and and *t* is the incubation time (s). Each measurement was performed in triplicate, data is presented as mean ± SD.

In parallel with HPLC, the determination of corneal permeability was investigated with Raman mapping after 15, 30 and 60 min treatment. The treated cornea was frozen and divided into cross Sects. (15 μm thick) with a Leica CM1950 Cryostat (Leica Biosystems GmbH, Wetzlar, Germany). Aluminum-coated slides were used under the 15-μm-thick cross-sections. Raman spectroscopic analysis was carried out with a Thermo Fisher DXR Dispersive Raman Spectrometer (Thermo Fisher Scientific Inc., Waltham, MA, USA) equipped with a CCD camera and a diode laser operating at 780 nm. Microscopic lens with 50 × magnification was used. Measurements were carried out with a laser power of 24 mW and a slit aperture of 50 μm. Cornea mapping was captured of an area of 150 × 1000 μm, with a step size of 50 μm vertically and horizontally. The OMNIC for Dispersive Raman 8.2 software (Thermo Fisher Scientific) was used for chemical evaluation. The individual spectrum of unformulated AA was used as a reference when profiling the chemical map.

### Statistical analysis

All data were presented as means ± SD. The normality of experimental data was determined by Kolmogorov–Smirnov test. In case of non-normal distributed data, as viscosity measurement results were analyzed with the non-parametric Kruskal–Wallis H test, whereas chemical stability, drug release and PAMPA permeability studies were compared using the non-parametric Mann–Whitney U test (TIBCO Statistica® 13.4, Statsoft Hungary, Hungary). In the case normal distributed ex vivo permeability measurement results paired *t*-test was applied. Changes were considered statistically significant at *p* < 0.05.

## Results and discussion

AA was encapsulated in liposomes by means of an easy and rapid method that did not involve the use of organic solvents. The main physicochemical characteristics of the liposome (AA-Lipo and LLipo) formulations, such as mean diameter, polydispersity and surface charge, were assessed. Light scattering results, summarized in Table 1, showed that AA-Lipo was small in size (~ 80 nm), with good homogeneity (P.I. 0.23), and negative zeta potential (~ − 10 mV). The filtration, which was performed to maintain sterility and prevent contamination of the samples, did not alter these values (*p* = 0.27; Table 1). To ensure the stability of the AA-Lipo on storage, the dispersions were lyophilized and easily re-dispersed in sterile water prior to use. After re-dispersion by gentle shaking, the size of the lyophilized liposome (AA-LLipo) formulation and the polydispersity increased (*p* = 0.007; Table 1), still falling within a size range that is advantageous for the intended application. The AA-LLipo samples were safely stored for three months and, once re-dispersed in water, maintained the above physicochemical features, but protection from light and moisture was an essential requirement to avoid oxidation of AA.

**Table 1:** Characteristics of liposomes loaded with ascorbic acid: mean diameter, polydispersity index (P.I.), zeta potential (ZP). Each value represents the mean ± SD, *n* = 300; ^**§**^ SD for P.I. values was ≤ 0.03.

Formulation	Mean diameter nm ± SD	P.I. ^§^	ZP mV ± SD
AA-Lipo	84 ± 4.4	0.23	− 9 ± 3.4
Filtered AA-Lipo	86 ± 2.1	0.23	− 10 ± 1.9
Re-dispersed AA-LLipo	101 ± 5.1	0.30	− 10 ± 2.2

AA, ascorbic acid; Lipo, liposomes; LLipo, lyophilized liposomes.

**Figure 1:**
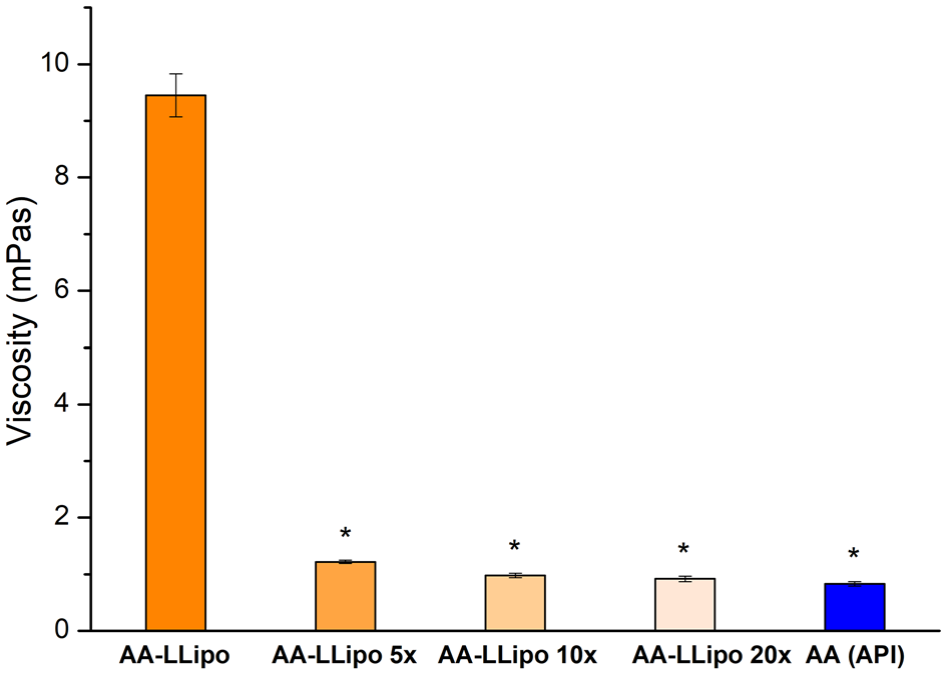
Effect of dilution (AA-LLipo 5 × , 10 × and 20 ×) on the viscosity of AA-LLipo formulation. Statistical analysis: Kruskal–Wallis H test. (*p = 0.012 compared to AA-LLipo). Data is presented as means ± SD, *n* = 6.

The effect of blinking and tearing on the rheological behavior of the AA-LLipo formulation can be represented by the investigation of diluted AA-LLipo formulations. The original AA-LLipo were diluted at three different value (fivefold, tenfold, and 20-fold) and their dynamic viscosities were determined and compared to the standard AA-API formula. In Fig. 1, it can be seen, that the smaller dilution value (5 ×) caused a significant decrease in viscosity of AA-LLipo (initial viscosity for AA-LLipo 9.45 ± 0.38 mPas and for AA-LLipo 5 × 1.22 ± 0.03 mPas). In the case of further dilution, the viscosity further decreased (for AA-LLipo 10 × 0.98 ± 0.04 and for AA-LLipo 20 × 0.92 ± 0.05 mPas) and each diluted AA-LLipo well approached the viscosity of standard AA-API solution (0.83 ± 0.04 mPas). Thus, even the freshly dripped liposomal formulation is able to mimic the rheological behavior of the classic eye drop and it is retained during blinking and tearing, which could be a promising aspect regarding the application of liposomal formula in clinical practice.

### Solution stability of AA and AA-LLipo

**Figure 2:**
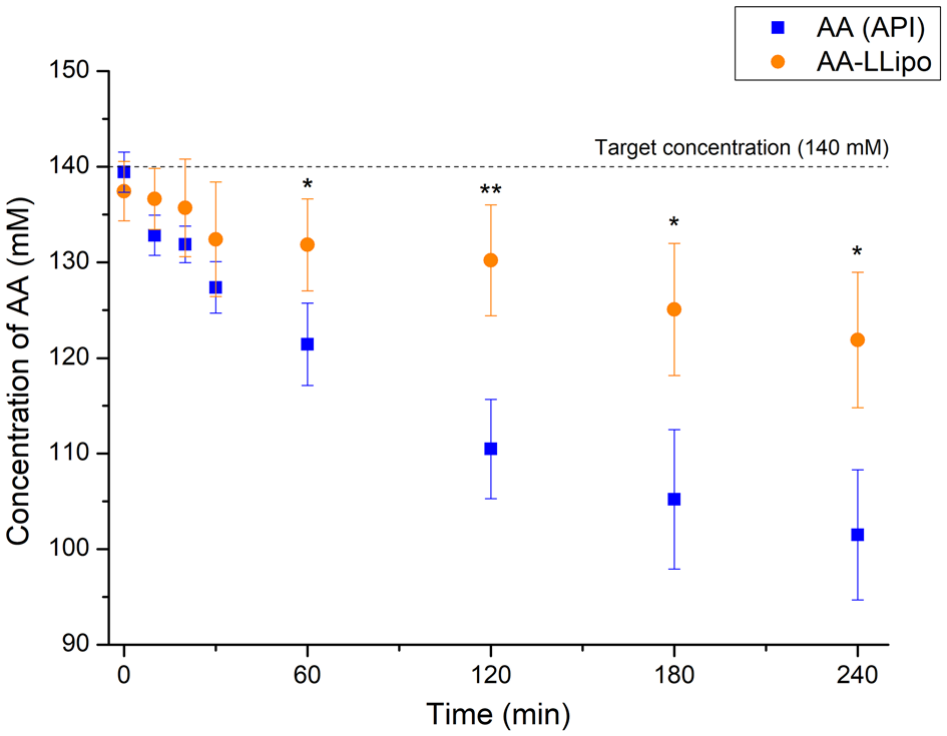
Chemical stability of AA (API) solution and AA-LLipo re-dispersed in pH 7.4 PBS at 35 °C. The chemical stabilities after 4 h were 77.0 and 89.8% for AA (API) and AA-LLipo, respectively. The shelf-life degradation (10%) level of AA (API) and AA-LLipo was observed at 79.3 and 209.6 min, respectively. Statistical analysis: Mann–Whitney U test. (*p = 0.032; **p = 0.0051 compared to AA (API) control in the corresponding time points). Data is presented as means ± SD, *n* = 5.

The chemical stability of AA (API) aqueous solution and AA-LLipo was investigated to model the normal degradation of AA during corneal-PAMPA permeability and RED release measurements. The stability results are shown in Fig. 2.

One of the limitations on the use of AA for topical or liquid formulation is its well-known chemical degradation^28, 29^. The development of a liposomal formula could be an effective solution, thus liposome-based formulation was chosen as starting point of our research. As it has previously been demonstrated, the degradation of AA can be effectively reduced in a liposomal system, due to the association with a hydrophilic-hydrophobic interface, which promotes the drug stability^30^. Accordingly, in a first step, a 4-h stability of AA solution and lyophilized liposomal AA (AA-LLipo) formulation diluted in PBS (pH 7.4) were compared at 35 °C, which corresponded to the incubation conditions of the subsequent in vitro permeability assay (corneal-PAMPA). It has been shown in Fig. 2, that the chemical stability of AA (API) solution after 60 min shows significant differences compared to the AA-LLipo formulation. Furthermore, while the AA (API) solution already reached shelf-life degradation level (10%) at 79.3 min, the shelf-life degradation for the AA-LLipo formulation was only observed at 209.6 min. The chemical stabilities –in 4-h timeframe according to incubation conditions of corneal-PAMPA, were 77.0% and 89.8% for AA (API) and AA-LLipo, respectively. The degradation kinetics parameters of the two formulas reported in Table 2 showed a good agreement with previous observations, i.e., the liposomal formula effectively stabilized AA in solution. Thus, it can be suitable for ophthalmic topical treatment, considering the residence time of the drug on the corneal surface. In addition, the stability associated with the storage conditions of the AA was also improved.

**Table 2:** Degradation kinetic parameters of AA and AA-LLipo.

Formulation	t (min)	C_0_/C_t_	log (C_0_/C_t_)	k (1/min)	t_1/2_ (min)	t_90%_ (min)
AA	240	1.37	0.138	0.0013	523.5	79.3
AA-LLipo	240	1.13	0.052	0.0005	1383.5	209.6

AA, ascorbic acid; LLipo, lyophilized liposomes; C_0_, concentration of AA at time point zero; C_t_, concentration of AA at time point t; k, rate constant of AA degradation process, t_1/2_, degradation half-life; t_90%_, shelf-life.

### Release of AA (API) and AA-LLipo

**Figure 3:**
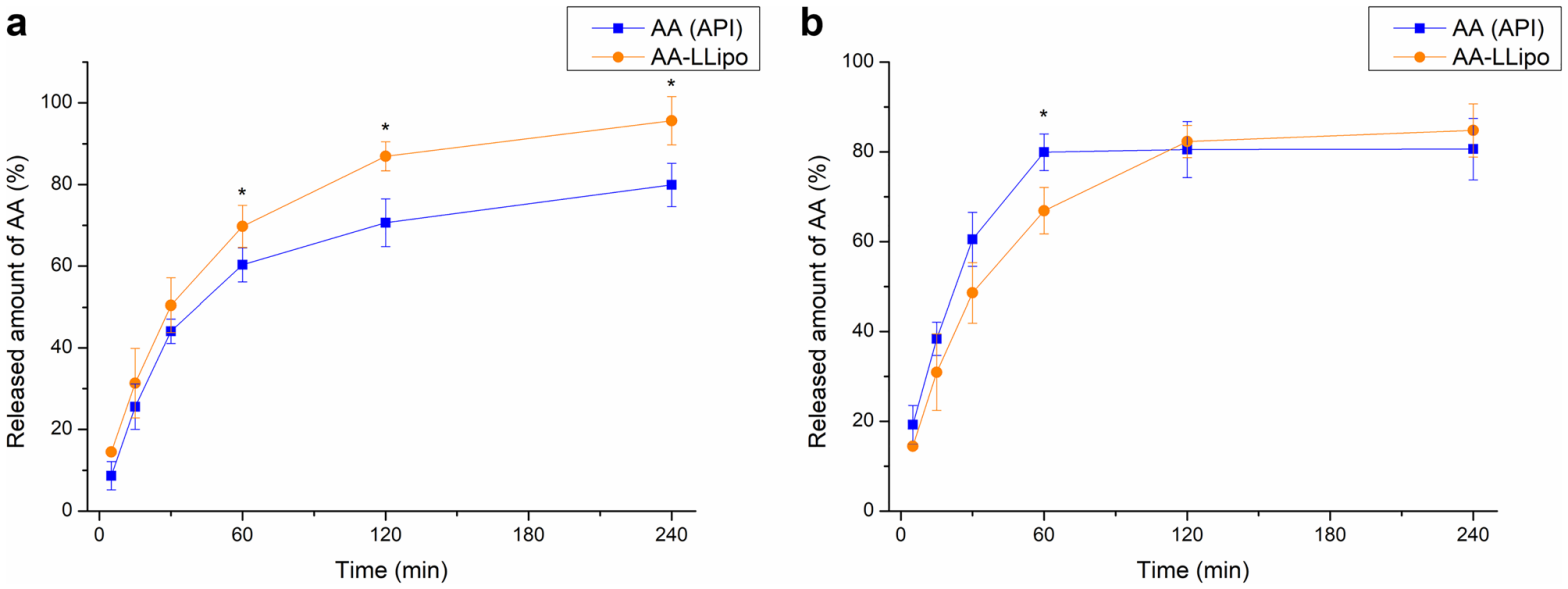
Drug release profiles of standard AA and AA-LLipo determined by RED method. Statistical analysis: Mann–Whitney U test. (**p = 0.0083 compared to AA (API) control in the corresponding time points) (**a**) and degradation kinetics based correction of drug release profiles (**b**). Data is presented as means ± SD, *n* = 5.

RED method was applied to determine the drug release of AA (API) solution and AA-LLipo. The drug release profiles can be seen in Fig. 3.

Similar to the chemical stability investigations, the release tests of the two AA formulas were determined using a rapid equilibrium dialysis (RED) system at 35 °C over a 4-h time interval. Based on the kinetic course of AA release shown in Fig. 3a, it can be seen that, the higher AA release from the AA-LLipo formulation starts to differ significantly from the standard AA solution a 60 min, which is in line with the result of the stability test. Drug release tests demonstrated that AA starts to degrade at 35 °C in the 4-h timeframe, however the degradation rate is lower in the case of encapsulating in liposomal carrier. This means liposomes are suitable for stabilization of AA in solution. The degradation kinetics with the actual drug concentration values can be plotted (Fig. 3b). Taking the degradation kinetics-based correction, the liposomal carrier showed moderate efficiency of drug release, moreover until 60 min, the release rate of AA (API) was higher in comparison to AA-LLipo formula, as expected. However, regarding the therapeutic applications, the degradation is a significant issue, which influences also the effectivity of the formulation. Therefore, a basic drug release results without degradation kinetics-based correction could represent a more realistic behavior of both formulas. Based on these considerations, the necessity of a liposomal carrier-based formula could be obviously realized.

### In vitro corneal-PAMPA study for AA (API) and AA-LLipo

**Figure 4:**
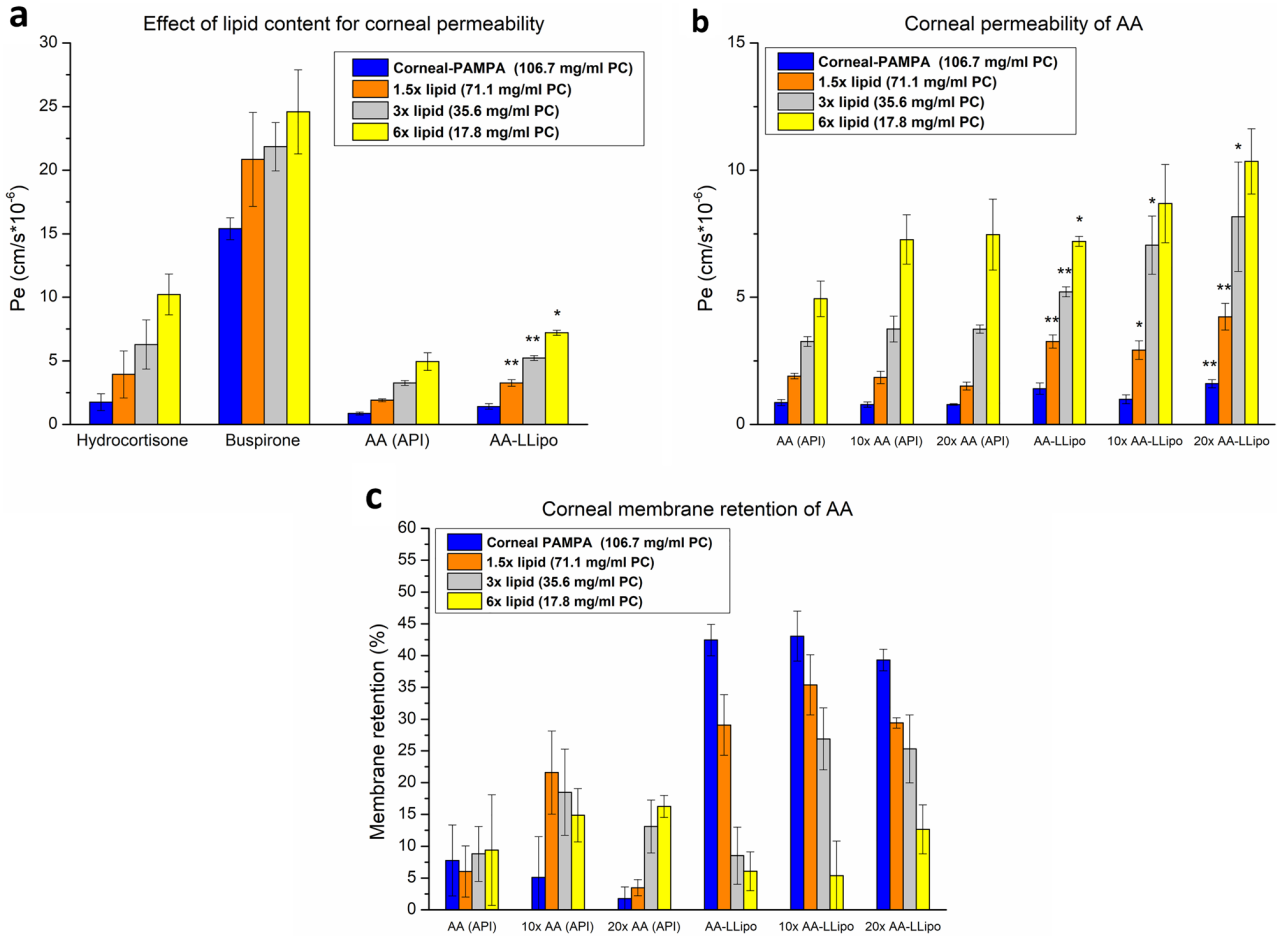
In vitro permeability assay using corneal specific PAMPA models (Lipid concentration in dodecane solution: corneal-PAMPA: 106.7 mg/mL, 1.5 × diluted: 71.1 mg/mL, 3 × diluted: 35.6 mg/mL, 6 × diluted: 17.8 mg/mL). Statistical analysis: Mann–Whitney U test. (*p = 0.025; **p = 0.0062 compared to AA (API) at corresponding lipid concentration). Data is presented as means ± SD, *n* = 6.

The characterization of the topical absorption of the liposomal formulation was performed in the corneal-PAMPA system developed by our research group, where healthy rabbit corneal penetration values of known drugs are used for the development of in vitro non-cellular permeability model^31^. However, changes in the integrity and enhanced penetration of partially damaged corneal epithelium during postoperative treatment and inflammatory processes have been reported in several studies^32–35^. Given the reduced permeability of AA due to its hydrophilic nature, which were wanted to be addressed by liposomal formulation, the modelling of the change in penetration in the healing process of the corneal epithelium was also aimed in this study. The decreasing behavior of corneal epithelium integrity in the corneal-PAMPA model can be represented by the various concentrations of lipid (PC) in a dodecane solution. The relationship between lipid concentration and drug permeability was identified during the development of our previous blood–brain barrier-specific PAMPA model ^36^. However, no thematic in vitro study was performed to model the penetration conditions of damaged or disrupted cornea. Consistent with this, the previously developed corneal-PAMPA model, whose artificial membrane was formed from a solution of 106.67 mg/mL PC in dodecane, was modified in three dilution steps for lipid concentrations (1.5×, 3×, and 6×). The effect of PC concentration on the permeability of corneal-PAMPA was performed in parallel with the PBS (pH 7.4) solution of the AA (API) and the re-dispersed AA-LLipo formulation, using the buspirone (BUS) and hydrocortisone (HYD) as a positive and negative control compounds, respectively. Based on our preliminary results presented in Fig. 4a, the increasing permeability corresponding to our basic hypothesis can be traced for both BUS and HYD. In case of all investigated PC concentration BUS showed significantly higher permeability as positive corneal PAMPA control in comparison to HYD. The trend-like change in permeability is consistent with in vivo studies that report increased permeability and drug transport conditions in the inflammatory and postoperative states of the cornea^32–35^. Similar results were obtained for the dilutions of AA (API) solution and AA-LLipo formulation. In the model corresponding to an intact corneal condition (corneal-PAMPA), reduced effective permeability values corresponding to the hydrophilic nature of AA were obtained, while decreasing the lipid (PC) concentration in the artificial membrane gave increasing permeability values nearly identical to the permeability of HYD as a negative control (Fig. 4a). Comparing the permeability conditions of the AA (API) and AA-LLipo, no significant difference was observed in the corneal-PAMPA model with elevated lipid concentration (normal corneal PAMPA), while with the decrease of PC concentration the increase in permeability of the AA-LLipo formulation was significantly greater in comparison to unformulated AA (API) solution. We also performed our in vitro corneal permeability model under the appropriate physiological conditions for topical ophthalmic application, in which the lacrimal fluid is able to significantly (approximately 20-fold) dilute the concentration of the drug on the surface of the eye^31^. This process can be linked to the defense mechanism of the eye with increased blinking and tearing. Accordingly, permeability assays were performed with tenfold and 20-fold dilutions (applying PBS, pH 7.4) of AA (API) solution and AA-LLipo on two scales. This was also necessary because of the ex vivo porcine eye studies performed later, only the AA (API) solution and AA-LLipo diluted 20-fold were used. Based on the results presented in Fig. 4b, a significant change in permeability upon dilution of AA formulations was identified for 3- and sixfold diluted lipid membrane systems. It can also be seen that although the permeability of both AA formulas increased with dilution of the base eye drop form, a larger improvement was observed with AA-LLipo. In close connection, dilution of the formulations also significantly increased the lipid membrane retention of the AA (Fig. 4c).

### Ex vivo porcine eye kinetic study

**Figure 5:**
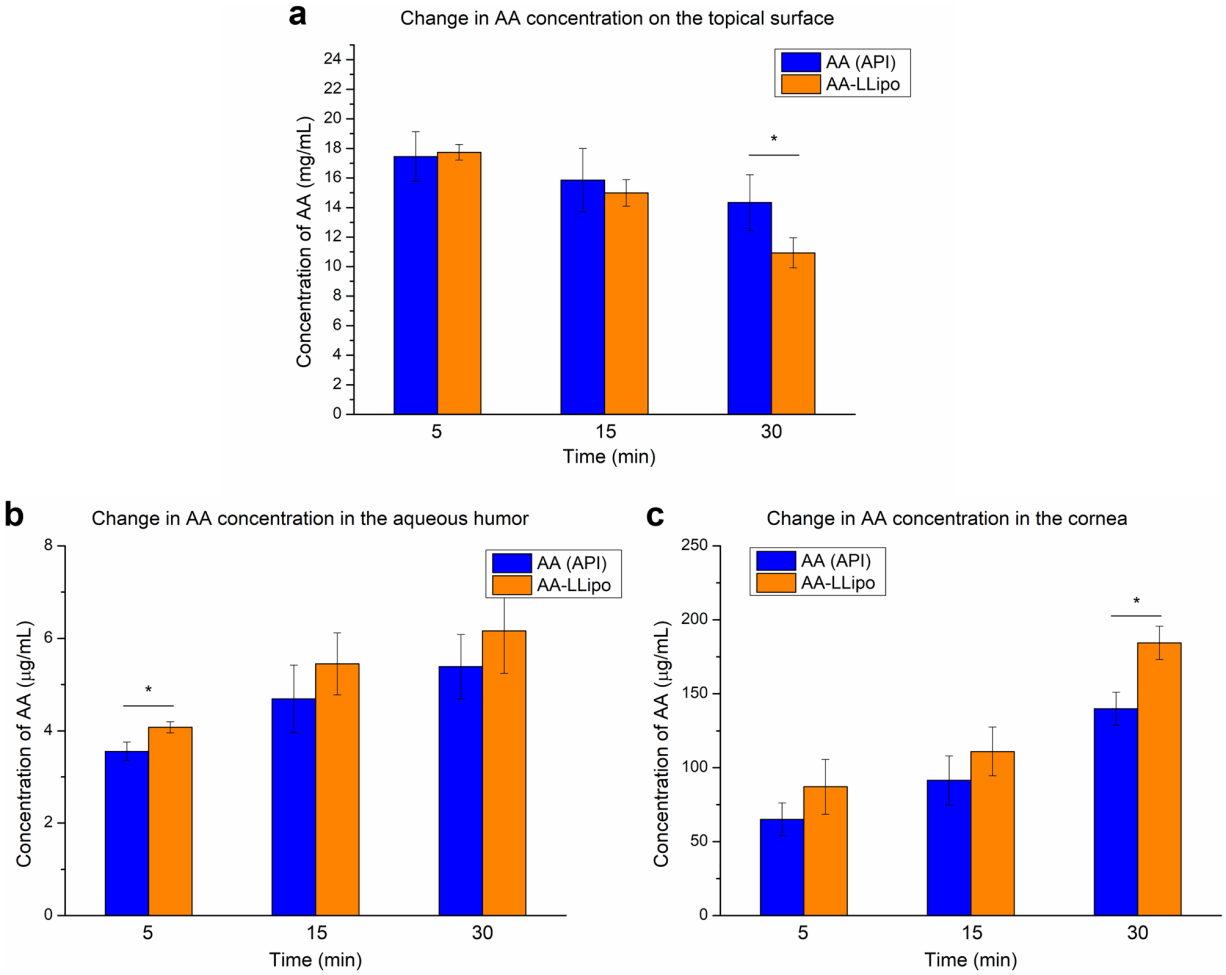
AA concentration at different eye’s compartments after treatment with 20-fold diluted AA solution and AA-LLipo formulation. Change in AA concentration on the topical surface (**a**), in the aqueous humor (**b**) and in the cornea (**c**). The surface concentration of AA decreasing implies that the liposomal cargo is penetrating into the target tissues more readily than free cargo (**a**). Statistical analysis: *t* test. (*p = 0.016 compared to AA (API) control in the corresponding time points). Data is presented as means ± SD, *n* = 3.

**Figure 6:**
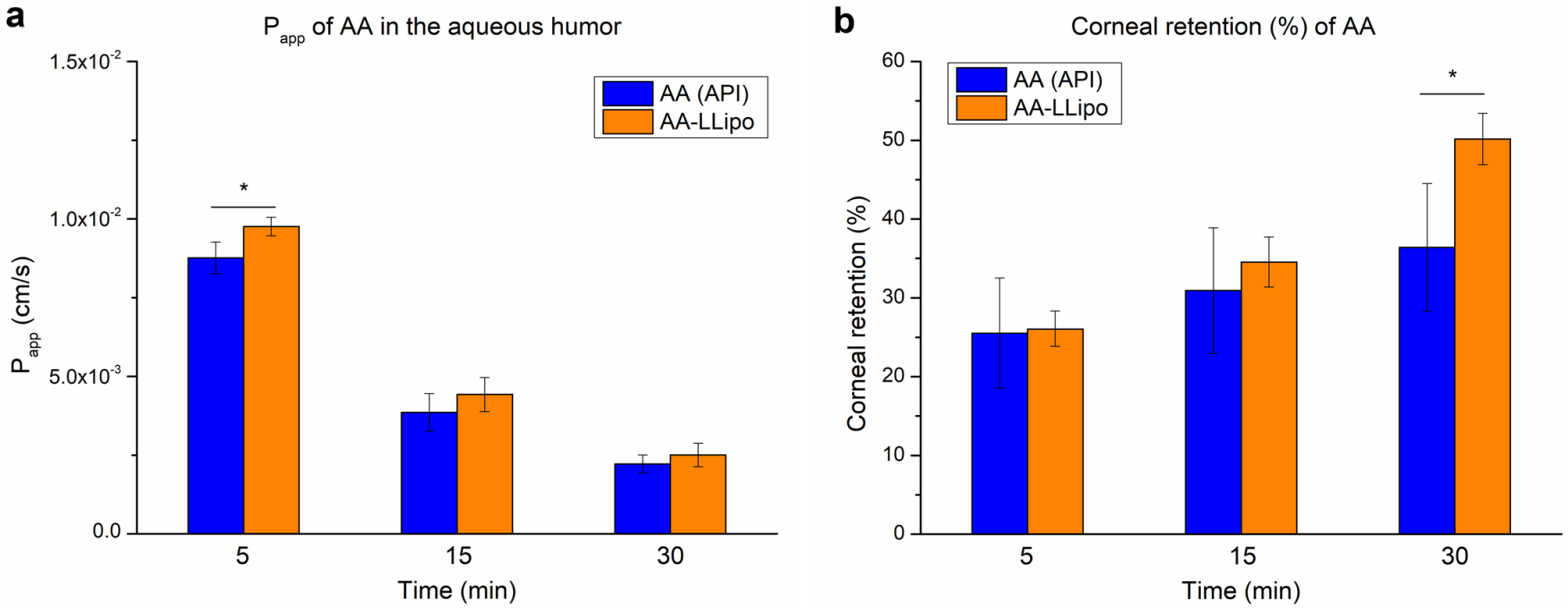
AA permeation (**a**) and corneal retention (**b**). Statistical analysis: *t* test. (*p = 0.017 compared to AA (API) control in the corresponding time points). Data is presented as means ± SD, *n* = 3.

The ocular absorption kinetics of the AA-LLipo formulation was also characterized by an ex vivo study in porcine eyes. Considering the expected rapid in vivo leaching (related to blinking and tearing) of AA due to its the hydrophilic character and the relatively narrow 1-h time scale for the ex vivo porcine eye study, the ex vivo kinetic studies were performed at 5, 15, and 30 min. As shown in Fig. 5, in a first approximation, the changes in AA concentration measured in pharmacokinetically relevant compartments (corneal surface, cornea, aqueous humor) were compared after treatment with 20-fold dilutions of the AA (API) solution and AA-LLipo formulation. Based on the results, the AA-LLipo formulation showed more favorable absorption properties at all kinetic time points on porcine eye compared to the AA (API) solution. In addition, a significantly greater decrease in AA concentration was observed at the eye surface and significantly greater AA accumulation was measured in the cornea at the last kinetic time point (after 30 min) for AA-LLipo (Fig. 5a and c). Furthermore, a slightly but significantly higher amount of drug was deposited in the aqueous humor at 5 min using AA-LLipo formulation. Overall, the increased AA concentration in the cornea (Fig. 5c) provides the most important information on the potential therapeutic benefit of the AA-LLipo formulation. In addition, the effective permeability (P_eff,ex vivo_) and corneal retention values of the diluted samples (Fig. 6a and b) were also calculated using the determined AA concentrations in each compartment. In parallel with the kinetic characteristics shown in Fig. 5, the AA-LLipo formulation provided higher average permeability and corneal retention of AA at all three time points. In the P_eff,ex vivo_ at 5 min and corneal retention at 30 min, a significant difference was identified between the 20-fold dilutions of the AA (API) solution and AA-LLipo formulation.

**Figure 7:**
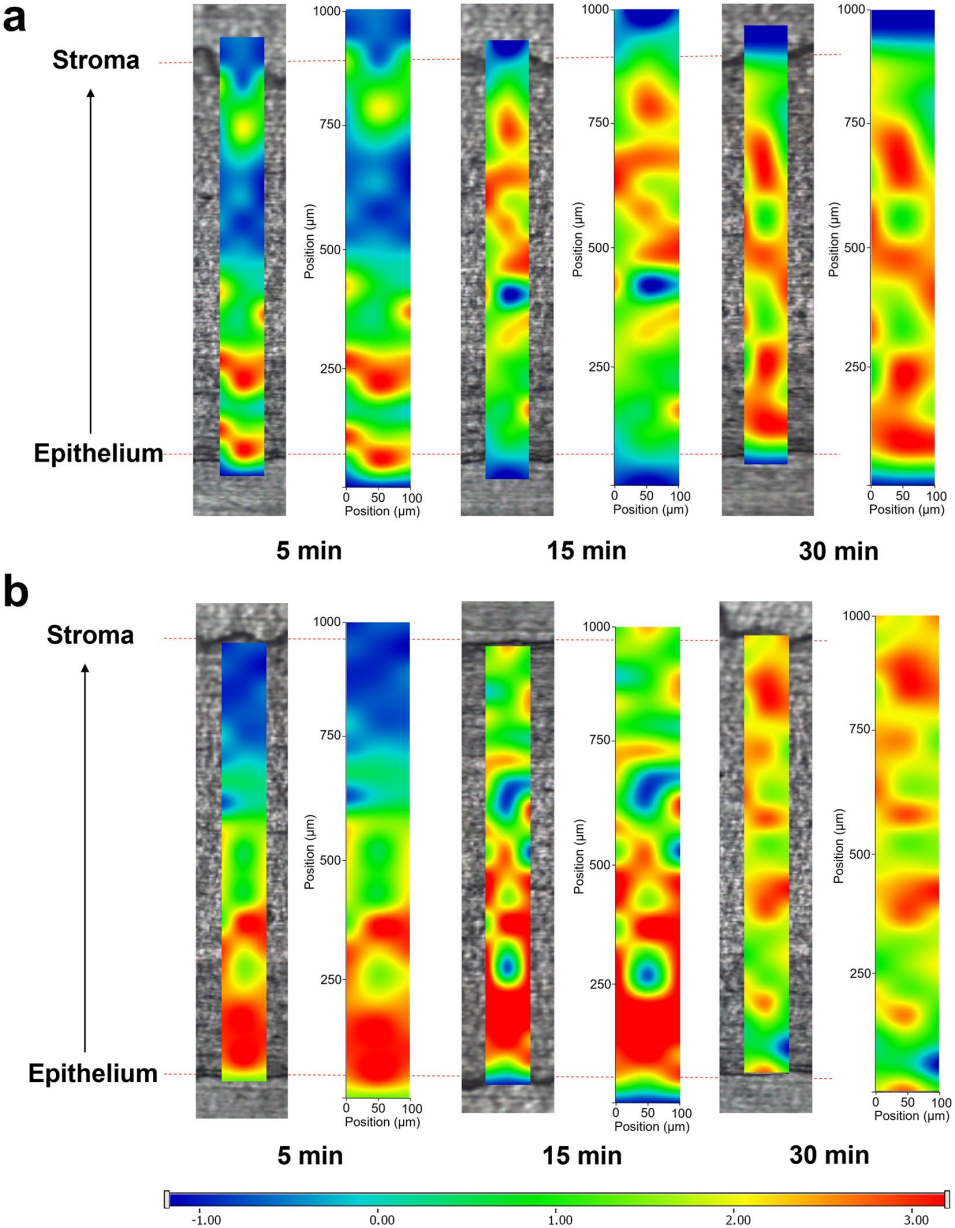
Raman maps of porcine cornea treated with AA (API) solution (**a**) and AA-LLipo formulation (**b**).

Raman maps of porcine corneal tissues treated with both AA solution and AA-LLipo are presented in Fig. 7. Red color in the chemical maps indicates the stronger existence of AA, the green area shows the presence of AA in lower concentration, whereas blue color marks those regions of the map, whose spectral resolution contains different spectra, characteristic of untreated corneal tissue. Results of the Raman mapping showed that after 5 min both AA solution (Fig. 7a) and AA-LLipo (Fig. 7b) penetrated into equal depth inside the cornea, no remarkable difference was observed. However, after 15 min AA (API) solution had already reached the inner part of corneal tissue, indicating faster penetration. After 30 min, AA-LLipo successfully penetrated into the corneal tissue reaching the stroma, which can be claimed with the controlled release property of liposomal carrier, while AA solution was concentrated in the epithelial part of cornea. This phenomenon can be explained by the lipophilic features of liposomal carrier, which improves the penetration and distribution of the water-soluble AA.

In conclusion, the developed liposomal AA formulation (AA-LLipo) was able to effectively increase the chemical stability of ascorbic acid in aqueous solution, which is also a significant advantage in terms of application and storage of eye drops. The release studies showed that AA can be released from the AA-LLipo formulation with the same kinetic efficiency as from the AA (API) solution, which can be an important expectation due to the relatively short residence time on the corneal surface. Our studies extended to the development of a previously introduced in vitro corneal permeability system (corneal-PAMPA), on the basis of which the test system became suitable for examining the absorption conditions of the postoperative and/or inflammatory state of the cornea. Both the modified in vitro corneal-PAMPA and ex vivo studies on porcine eyes showed that AA-LLipo can greatly improve the pharmacokinetic properties of ascorbic acid related to the eye, thus significantly increasing the accumulation of AA in the cornea (lipid membrane retention: corneal-PAMPA, corneal retention: ex vivo study) and can simultaneously increase its permeability and concentration in the aqueous humor. The enhanced corneal AA content reached with the topical administration of the AA-LLipo formulation might be a new therapeutic option for preventing corneal scarring after ocular surface surgeries, injuries and inflammations. The effect of the chemical composition, morphology and internal structure of nanostructured systems can open new opportunities for enhancing the efficiency of many biologically active compound for ophthalmic uses. Future studies are needed regarding the effect of liposomal AA on corneal scarring in vivo.

## Data Availability

The datasets used and/or analyzed during the current study are available from the corresponding authors on reasonable request.
